# Associations between maternal mental health and early child wheezing in a South African birth cohort

**DOI:** 10.1002/ppul.24008

**Published:** 2018-04-10

**Authors:** Rae P. MacGinty, Maia Lesosky, Whitney Barnett, Dan J. Stein, Heather J. Zar

**Affiliations:** ^1^ Department of Pediatrics and Child Health Red Cross War Memorial Children's Hospital and Medical Research Council Unit on Child and Adolescent Health University of Cape Town Cape Town South Africa; ^2^ Division of Epidemiology and Biostatistics School of Public Health and Family Medicine University of Cape Town Cape Town South Africa; ^3^ Department of Psychiatry and Mental Health Medical Research Council Unit on Anxiety and Stress Disorders and Medical Research Council Unit on Risk and Resilience in Mental Disorders University of Cape Town Cape Town South Africa

**Keywords:** antenatal, intimate partner violence, low‐ and middle‐income countries, maternal depression, postnatal, psychological distress, wheeze

## Abstract

**Background:**

Wheezing in early childhood is common and has been identified in high‐income countries (HIC) as associated with maternal antenatal or postnatal psychosocial risk factors. However, the association between maternal mental health and childhood wheezing has not been well studied in low and middle‐income countries (LMIC), such as South Africa.

**Methods:**

A total of 1137 pregnant women over 18 year old, between 20 and 28 weeks’ gestation, and attending either of two catchment area clinics were enrolled in a South African parent study, the Drakenstein Child Health Study (DCHS). Psychosocial risk factors including maternal depression, psychological distress, early adversity, and intimate partner violence (IPV), were measured antenatally and postnatally by validated questionnaires. Two outcomes were evaluated: Presence of wheeze (at least one episode of child wheeze during the first 2 years of life); and recurrent wheeze (two or more episodes of wheezing in a 12‐month period). Logistic regression was used to investigate the association between antenatal or postnatal psychosocial risk factors and child wheeze, adjusting for clinical and socio‐demographic covariates.

**Results:**

Postnatal psychological distress and IPV were associated with both presence of wheeze (adjusted OR = 2.09, 95%CI: 1.16‐3.77 and 1.63, 95%CI: 1.13‐2.34, respectively), and recurrent child wheeze (adjusted OR = 2.26, 95%CI: 1.06‐4.81 and 2.20, 95%CI: 1.35‐3.61, respectively).

**Conclusion:**

Maternal postnatal psychological distress and IPV were associated with wheezing in early childhood. Thus, screening and treatment programs to address maternal psychosocial risk factors may be potential strategies to reduce the burden of childhood wheeze in LMICs.

## 1 INTRODUCTION

Wheezing in early childhood is very common, with 50% of children from high‐income countries (HIC) reported to have experienced an episode of wheezing before 6 years of age.^1^ Wheezing illness comprises a spectrum of disease, ranging from transient to recurrent, a proportion of which is associated with asthma.^2^ Asthma is the most common chronic illness in children, and particularly high in Africa; thus, it is important to understand the risk factors associated with wheeze onset.^2^ There are many causes of wheezing in early childhood and several risk factors associated with the development or severity of wheezing. The most common risk factors include environmental tobacco smoke (ETS) exposure; genetic predisposition; early viral lower respiratory tract infections (LRTI); low socio‐economic status and poor living conditions; as well as an increased risk in males.^3^ A more recent focus is on maternal psychosocial exposures and the impact these have on child wheeze development and recurrence.

Antenatal or postnatal maternal psychosocial risk factors have been reported to be associated with development of child wheeze,^4^, ^5^, ^6^, ^7^, ^8^, ^9^, ^10^, ^11^, ^12^, ^13^ but there is sparse data from low and middle‐income countries (LMIC). Most research has been conducted in HIC, and predominantly in high‐risk populations. These results provide valuable insight into the relationship between maternal mental health and respiratory outcomes in children, but unique genetic and cultural factors may impact associations in LMIC differently than HIC. This study investigated the association between antenatal and postnatal maternal psychosocial risk factors and child wheeze in South Africa, addressing key gaps in the literature by expanding prior research to a LMIC in a generalizable population.

## 2 METHODS

### 2.1 Setting

This study was a sub‐study of the Drakenstein Child Health Study (DCHS), a multidisciplinary birth cohort investigating the epidemiology and etiology of childhood respiratory illness and the early life determinants of child health in a peri‐urban area in Paarl, South Africa.^14^ The catchment population is approximately 200 000, consisting mainly of those with low socio‐economic status, who reside in informal settings or crowded conditions.^14^, ^15^ More than 90% of the population access public healthcare services for their primary care.^14^

### 2.2 Participants

Participants were those enrolled in the DCHS. Inclusion criteria were women 18 years or older, who were at 20‐28 weeks’ gestation, attended one of two local clinics, provided written informed consent and intended to remain in the area for at least 1 year.^16^ Women were followed through childbirth and mother‐child pairs were followed through childhood.

### 2.3 Design

The birth cohort recruited pregnant women attending one of two primary healthcare clinics: Mbekweni, which predominately served a population with African‐ancestry and TC Newman which mostly served a mixed‐ancestry population.^15^ Child clinical and respiratory symptom questionnaires were completed at each of the study visits, which occurred at birth, 6‐10 and 14 weeks and 6, 12, 18, and 24 months post‐delivery at primary healthcare clinics.

### 2.4 Measures

Risk factor and outcome data collection is ongoing and recorded longitudinally as part of the DCHS. The primary outcome of this study was child wheeze through 2 years of age.

#### 2.4.1 Wheeze outcomes

Child wheeze was measured through maternal report at each of the study visits, as well as episodes identified through the active surveillance for respiratory symptoms associated with lower respiratory tract infections (LRTI). Active surveillance was performed by nurses at the primary clinics and assessed in real time.^14^, ^17^ These nurses were trained in respiratory examination of children and had to attend frequent competency assessments.^17^ Measurements of LRTI included ambulatory and hospitalized pneumonia cases, as defined by World Health Organization (WHO) criteria.^14^, ^17^, ^18^ As the mothers were interviewed frequently, it was also possible to retrospectively capture respiratory events occurring at other facilities or outside the area.^17^ Any information on respiratory events captured outside of the clinics was obtained by review of medical records.^17^

Two binary outcome variables were considered: Whether the child experienced at least one episode of wheeze during the first 2 years of life, or whether the child experienced recurrent wheeze episodes (2 or more wheeze episodes in a 12‐month period). Wheeze was considered present if it was reported during any routine study visit or identified by study staff when examining the child at a LRTI visit in the first 2 years of life.

#### 2.4.2 Maternal psychosocial measures

Maternal psychosocial data was collected antenatally, and postnatally at 6‐10 weeks and 6, 12, 18, 24 months postpartum.^15^ Several validated questionnaires were used to measure psychosocial risk factors: The Edinburgh Postnatal Depression Scale (EPDS) is a widely used and reliable measure of depressive symptoms and was used to measure maternal depression.^19^ Each of 10 questions were scored 0‐3 and totalled.^15^ A cut‐off value of 13 was used to separate the participants into above‐ or below‐threshold groups.^19^, ^20^

The Self‐Reporting Questionnaire 20‐item (SRQ20),^21^ a widely used and validated measure, was used to determine the presence of maternal psychological distress.^22^, ^23^ Each item was scored 0‐1, and a total score generated.^15^ A cut‐off value of 8 dichotomized participants into an above‐ or below‐threshold group.^15^, ^22^, ^24^

The Intimate Partner Violence (IPV) Questionnaire was used to assess maternal physical, emotional and sexual violence exposure.^25^, ^26^ Exposure to IPV was dichotomized by those recently experiencing any one of the three violent exposures versus no exposure.

Other psychosocial measures included: the Childhood Trauma Questionnaire, to assess childhood abuse and neglect,^15^, ^27^ which was dichotomized into above‐ or below‐threshold based on any exposure versus no exposure; the Modified Post‐Traumatic Stress Disorder Symptom Scale used to screen for current post‐traumatic stress disorder (PTSD),^28^ which was categorized into three mutually exclusive levels (no exposure, trauma exposed and suspected PTSD).

#### 2.4.3 Clinical and sociodemographic data

Covariates considered for the analyses included: child feeding practices; HIV exposure; maternal smoking, through self‐report, and environmental tobacco smoke (ETS) exposure, assessed by the number of smokers in the child's household; alcohol consumption during pregnancy, measured by the Alcohol, Smoking and Substance Involvement Screening Test (ASSIST)^29^; maternal or family history of asthma ascertained by maternal report; birth characteristics, such as gestational age and birth weight, measured by study staff; child vaccination; socio‐economic status (SES) based on a composite score considering four socio‐economic variables: level of education, employment status, household income, and number of asset.^30^ Standardized scores were divided into quartiles, which included “low,” “low‐moderate,” “high‐moderate,” and “high” groups. A time variable, in months, was also generated to measure a child's follow‐up time throughout the 24‐month period.

### 2.5 Ethical considerations

The DCHS was approved by the Faculty of Health Sciences, Human Research Ethics Committee (HREC), University of Cape Town (401/2009), and by the Western Cape Provincial Health Research committee.^14^ Mothers provided written informed consent, which was voluntary and renewed annually.

The current study was approved by HREC (Ref number: 387/2017).

### 2.6 Statistical analysis

Analyses were conducted with STATA version 13.0 (College Station, TX). Descriptive data was presented as medians, interquartile ranges (IQR) and frequencies (proportions), as appropriate. Mann‐Whitney rank sum and Kruskal‐Wallis tests were used to test for associations between categorical and continuous variables, as all continuous variables were nonparametric. Pearson Chi‐square test or Fisher Exact tests were used to determine if significant associations existed between categorical variables.

Multivariable logistic regression was used to model the associations of maternal psychosocial risk factors, both antenatally and postnatally, with the occurrence and recurrence of child wheezing, adjusting for confounding clinical and sociodemographic covariates. As multicollinearity was present among the psychosocial risk factors, they were considered individually in a series of logistic regression models. The postnatal psychosocial risk factor measures were also found to be correlated over time, thus we utilized data from the 6‐month scheduled visit as a proxy for postnatal exposure, as most wheezing episodes occurred within the first 6 months of life.

Diagnostic checks were generated for all multivariable models. Based on Pearson's chi‐squared and/or the Hosmer‐Lemeshow test, all the models were found to correctly specify the association between perinatal maternal psychosocial risk factors and child wheezing outcomes.

## 3 RESULTS

### 3.1 Descriptive statistics and exploratory analysis

In total, 1137 women with 1143 Live births, were enrolled in this study, Supplementary Figure S1. At the end of the 2‐year follow‐up, 985 children were still active in the study, and the total child follow‐up time was 1859.54 years.

#### 3.1.1 Socio‐demographics and clinical factors

The median maternal age was 26 (22.3‐31.1) years; 22% of the women were HIV‐infected, with a significantly higher prevalence (37%) of HIV in the Mbekweni participants compared to those of TC Newman (3%), Table 1. Approximately 27% of the women smoked during pregnancy, with the majority (53%) from TC Newman. In addition, a higher number of household smokers, and antenatal maternal alcohol consumption, were reported in TC Newman relative to those attending Mbekweni, Table 1.

**Table 1:** Study participant characteristics

	All participants (*N* = 1143 children; 1137 mothers)	Participants from Mbekweni (*N* = 634)	Participants from TC Newman (*N* = 509)	*P*‐value
Socio‐demographics				
Females	554 (48.5%)	322 (50.8%)	232 (45.6%)	0.080
Race: African	632 (55.3%)	626 (98.7%)	6 (1.2%)	<0.0001
Race: Mixed‐ancestry	511 (44.7%)	8 (1.3%)	503 (98.8%)	‐
Household income per month (South African Rand [ZAR])^*^				
<R1000	420 (36.7%)	262 (41.3%)	158 (31.2%)	<0.0001
R1000‐R5000	565 (49.5%)	305 (48.1%)	260 (51.3%)	‐
>R5000	156 (13.7%)	67 (10.6%)	89 (17.6%)	‐
Education				
Primary	86 (7.6%)	49 (7.7%)	37 (7.3%)	0.045
Secondary	609 (53.2%)	343 (54.2%)	266 (52.2%)	‐
Completed secondary	375 (32.8%)	192 (30.0%)	183 (36.0%)	‐
Any tertiary	73 (6.4%)	50 (7.9%)	23 (4.5%)	‐
Socioeconomic status quartile				
Lowest	274 (23.9%)	185 (29.2%)	89 (17.5%)	< 0.0001
Low‐moderate	296 (25.9%)	174 (27.4%)	122 (24.0%)	‐
High‐moderate	290 (25.4%)	146 (23.0%)	144 (28.3%)	‐
Highest quartile	283 (24.8%)	129 (20.3%)	154 (30.2%)	‐
Maternal or household characteristics				
Maternal age at delivery	26.07 (22.29‐31.09)	27.01 (22.66‐31.96)	24.99 (21.73‐29.45)	<0.0001
Antenatal maternal smoking	306 (26.8%)	39 (6.2%)	267 (52.5%)	<0.0001
Postnatal household smokers				
1	396 (34.8%)	230 (36.5%)	166 (32.7%)	<0.0001
>2	369 (32.4%)	113 (17.9%)	256 (50.4%)	‐
Antenatal Maternal Alcohol use^***^				
Low risk	886 (89.0%)	503 (93.2%)	383 (84.2%)	<0.0001
Moderate risk	78 (7.8%)	12 (2.2%)	66 (14.5%)	‐
High risk	31 (3.1%)	25 (4.6%)	6 (1.3%)	‐
Maternal HIV	248 (21.7%)	232 (36.6%)	16 (3.1%)	<0.0001
Family history of asthma	25 (2.2%)	6 (0.9%)	19 (3.7%)	0.001
Birth characteristics				
Gestation (weeks)	39 (38‐40)	39 (38‐40)	39 (37‐40)	0.287
Prematurity (<37 weeks)	194 (16.9%)	110 (17.4%)	84 (16.4%)	0.705
Birthweight (kg)	3.09 (2.71‐3.42)	3.16 (2.79‐3.46)	3.00 (2.63‐3.36)	<0.0001
Feeding choice				
Initiated breastfeeding	1 047 (91.6%)	542 (85.5%)	505 (99.2%)	<0.0001
Exclusive breastfeeding months	1.00 (0.45‐3.00)	0.92 (0.00‐2.76)	1.61 (0.92‐3.00)	<0.0001
Child follow‐up time (months)	23.98 (17.87 ‐25.00)	22.55 (16.82‐24.90)	24.57 (18.07‐25.07)	<0.0001
Vaccinations				
BCG (Birth)	1009/1016 (99.3%)	547/551 (99.3%)	462/465 (99.4%)	0.877
DTaP‐IPV‐Hib (6‐10 weeks)	981/986 (99.5%)	542/545 (99.4%)	439/441 (99.5%)	0.831
DTaP‐IPV‐Hib (10 weeks)	967/972 (99.5%)	532/535 (99.4%)	435/437 (99.5%)	0.823
DTaP‐IPV‐Hib (14 weeks)	943/952 (99.2%)	521/525 (99.4%)	422/427 (98.8%)	0.315
DTaP‐IPV‐Hib (18 months)	536/655 (81.8%)	323/342 (94.4%)	213/313 (68.1%)	< 0.0001
PCV13 (6‐10 week)	983/987 (99.6%)	543/546 (99.5%)	440/441 (99.8%)	0.428
PCV13 (14 week)	945/952 (99.3%)	522/525 (99.4%)	423/427 (99.1%)	0.512
PCV13 (9 months)	868/881 (98.5%)	492/496 (99.2%)	376/385 (97.7%)	0.062
Wheeze episodes				
Total Wheeze episodes^a^, ^b^, ^c^	924	420 (45.5%)	504 (54.5%)	‐
Total maternal reported episodes	437	152 (34.7%)	285 (65.3%)	‐
Total LRTIassociated episodes	0487	268 (55.0%)	219 (45.0%)	‐
No. of children with wheeze	479 (41.9%)	232 (36.6%)	247 (48.5%)	<0.0001
No. of children with recurrent wheeze	186 (16.3%)	78 (12.3%)	108 (21.2%)	<0.0001

HIV, human immunodeficiency virus; BCG, bacillus Calmette‐Guérin vaccine; DTaP‐IPV‐HiB, diphtheria, tetanus, acellular pertussis, polio, and haemophilus influenzae type b; PCV, pneumococcal conjugate vaccine; LRTI, lower respiratory tract infection.

^*^ Missing data from two participants from TC Newman.

**Missing data for six participants: three from Mbekweni and three from TC Newman.

^***^ Missing data from one‐hundred and forty‐eight participants: ninety‐four from Mbekweni and fifty‐four from the TC Newman.

^a^ wheeze episodes over time: 0‐6 months: *n* = 452; 712 months: *N* = 229; 13‐18 months: *N* = 151; 19‐24 months: *n* = 92.

^b^ Crude incidence rate: 0.497.

^c^ Incidence rate over 12‐ month period: 0‐12 months: 0.6998; 13–‐24 months: 0.2839.

Socio‐economic status (SES) varied between the two sites, with Mbekweni participants having a higher proportion of low SES households; overall approximately 86% of participants lived in households that earned less than 5000 South African Rand (ZAR) (416 USD) a month, Table 1.

#### 3.1.2 Birth characteristics

An even distribution of males and females were born; a small proportion (17%) of births were premature (<37 weeks’ gestation). Median birth weight was approximately 3 kg. Approximately 92% of the children had initiated breastfeeding, with a median duration of exclusive breastfeeding of 1 month during the follow‐up period, Table 1. Two (0.17%) of the children were HIV positive.

#### 3.1.3 Wheezing episodes

In total, there were 924 wheeze episodes (crude incidence rate = 497 cases per 1000 person‐years of follow‐up time) throughout the 24‐month follow‐up period, most of which occurred in those children attending TC Newman (55%). Mother or care‐giver reported wheeze contributed 437 (47%) episodes and the remainder (*n* = 484) were reported or identified during the active surveillance, and thus associated with LRTI. As much of the wheezing was associated with LRTI, it was not considered as an individual covariate. In addition, a high proportion of episodes occurred within the first 6 months of life (*n* = 452, 49%). At least one episode of wheeze was experienced by 479 (42%) children during the 24‐month follow‐up period, while 186 (16%) had recurring wheezing episodes.

#### 3.1.4 Psychosocial risk factors

##### Antenatal

Antenatal depression was present in 24% of the women (*n* = 237) with similar distribution across the sites. In addition, approximately 20% suffered from antenatal psychological distress, Table 2. There was a high prevalence of antenatal IPV in the study, with approximately 34% of participants being recently exposed; this was significantly different between the two sites, with the majority of exposure reported by those attending TC Newman. Exposure to maternal childhood trauma was also higher in those attending TC Newman (41%). However, PTSD was more common in Mbekweni relative to TC Newman.

**Table 2:** Antenatal and postnatal psychosocial risk factors

	All participants	Participants from Mbekweni	Participants from TC Newman	*P*‐value
Antenatal maternal psychosocial risk factors (*n* = 1003)				
Depression	237 (23.6%)	124/540 (23.0%)	113/457 (24.7%)	0.515
Psychological distress	203 (20.2%)	93/545 (17.1%)	110/458 (24.0%)	0.006
IPV (recent)^a^,^b^	332 (33.5%)	149/537 (27.7%)	183/455 (40.2%)	<0.0001
PTSD—Suspected PTSD (subset: *N* = 990)^c^	129 (13.0%)	92/536 (17.2%)	37/454 (8.1%)	<0.0001
PTSD—Trauma exposed (subset: *N* = 990)	123 (12.5%)	67/536 (12.5%)	56/454 (12.3%)	‐
Maternal childhood trauma	343 (34.2%)	155/545 (28.4%)	188/458 (41.0%)	<0.0001
Postnatal maternal psychosocial risk factors (6‐10 weeks) (*N* = 692)				
Depression	119 (17.2%)	62/366 (16.9%)	57/325 (17.5%)	0.835
Psychological distress	69 (10.0%)	18/366 (4.9%)	51/325 (15.7%)	<0.0001
IPV (recent)	177 (26.6%)	78/367 (21.3%)	99/325 (30.5%)	0.006
PTSD— suspected PTSD (subset: *N* = 198)	9 (4.6%)	8/126 (6.3%)	1/72 (1.4%)	0.326
PTSD—Trauma exposed (subset: *N* = 198)	10 (5.1%)	6/126 (4.8%)	4/72 (5.6%)	‐
Postnatal maternal psychosocial risk factors (6 months) (*N* = 645)				
Depression	97 (15.0%)	28/326 (8.6%)	69/319 (21.6%)	<0.0001
Psychological distress	61 (9.5%)	5/324 (1.5%)	56/319 (17.6%)	<0.0001
IPV (recent)	184 (28.6%)	69/324 (21.3%)	115/319 (36.1%)	<0.0001
PTSD—suspected PTSD (subset: *N* = 253)	23 (9.1%)	20/150 (13.3%)	3/103 (2.9%)	0.014
PTSD—Trauma exposed (subset: *N* = 253)	16 (6.3%)	9/150 (6.0%)	7/103 (6.8%)	‐
Postnatal maternal psychosocial risk factors (12 months) (*N* = 754)				
Depression	116 (15.4%)	36/376 (9.6%)	80/341 (23.5%)	<0.0001
Psychological distress	70 (9.3%)	11/399 (2.8%)	59/355 (16.6%)	<0.0001
IPV (recent)	199 (26.8%)	68/388 (17.5%)	131/354 (37.0%)	<0.0001
PTSD— suspected PTSD (subset: *N* = 55)	3 (5.5%)	0/10 (0.0%)	3/45 (6.7%)	0.771
PTSD— Trauma exposed (subset: *N* = 55)	4 (7.3%)	0/10 (0.0%)	4/45 (8.9%)	‐
Postnatal maternal psychosocial risk factors (18 months) (*n* = 663)				
Depression	65 (10.1%)	22/347 (6.3%)	43/294 (14.6%)	0.001
Psychological distress	53 (8.0%)	7/376 (1.9%)	46/296 (15.5%)	<0.0001
IPV (recent)	144 (21.7%)	62/367 (16.9%)	82/296 (27.7%)	0.001
PTSD—suspected PTSD (subset: *N* = 235)	18 (7.7%)	16/108 (14.8%)	2/127 (1.6%)	<0.0001
PTSD— Trauma exposed (subset: *N* = 235)	9 (3.8%)	5/108 (4.6%)	4/127 (3.2%)	‐
Postnatal maternal psychosocial risk factors (24 months) (*n* = 670)				
Psychological distress	51 (7.6%)	10/384 (2.6%)	41/286 (14.3%)	<0.0001
IPV (recent)	144 (21.5%)	69/383 (18.0%)	75/286 (26.2%)	0.011
PTSD— suspected PTSD (subset: *N* = 281)	16 (5.7%)	10/138 (7.2%)	6/143 (4.2%)	0.153
PTSD—Trauma exposed (subset: *N* = 281)	6 (2.1%)	1/138 (0.7%)	5/143 (3.5%)	‐

IPV, intimate partner violence; PTSD, post‐traumatic stress disorder.

^a^ IPV antenatal lifetime exposure *N* = 453 (46%).

^b^ Recent IPV considers incidents within the last 12 months.

^c^ PTSD questionnaire only given to those that have experienced a traumatic event.

##### Postnatal

A relatively consistent level of exposure of maternal depression, psychological distress and IPV existed across the scheduled follow‐up visits, Table 2. Based on Chi‐Squared Tests of Independence (Supplementary Table S1‐S4), high levels of correlation existed among the exposures. The prevalence of depression, psychological distress and IPV exposures were greater in those attending TC Newman compared to those attending Mbekweni.

### 3.2 Univariate and multivariable analysis

#### 3.2.1 Antenatal psychosocial risk factors and presence of child wheeze

Table 3 displays the associations with presence of wheeze and antenatal psychosocial risk factors. Due to collinearity, each antenatal psychosocial risk factor was included separately in a multivariable model, adjusting for key covariates as described. Maternal smoking, number of household smokers, and clinic attended were significantly associated with an increased risk for wheeze; whereas gestational age, and birth weight were found to have a protective association. Full‐term births and those with higher birth weight were less at risk of experiencing a wheezing episode.

**Table 3:** Logistic regression: antenatal psychosocial exposures and at least one episode of child wheeze

Predictors of at least one episode of child wheeze, with antenatal psychosocial risk factors as primary exposures		
Variable	Univariate OR (95%CI)	Multivariable OR (95%CI)
Antenatal maternal depression	1.12 (0.83, 1.50)	1.10 (0.81, 1.50)^1^
Antenatal maternal psychological distress	1.29 (0.94, 1.76)	1.20 (0.87, 1.67)^2^
Antenatal maternal IPV	1.06 (0.81, 1.38)	0.98 (0.74, 1.30)^3^
Antenatal maternal childhood trauma	1.22 (0.94, 1.59)	1.09 (0.82, 1.44)^4^
Antenatal maternal PTSD		
No PTSD exposure	REFERENCE	REFERENCE
Suspect PTSD	1.36 (0.94, 1.99)	1.59 (1.06, 2.36)^5^
Trauma exposed	0.89 (0.60, 1.31)	0.96 (0.64, 1.44)^5^
Maternal age at delivery	0.99 (0.97, 1.01)	‐
Maternal HIV status	1.02 (0.76, 1.35)	‐
Maternal smoking	1.68 (1.29, 2.18)	1.26 (0.91, 1.74)^6^
Number of postnatal household smokers		
None	REFERENCE	‐
1 person	1.45 (1.09, 1.94)	1.27 (0.94, 1.73)^6^
2 or more people	1.67 (1.24, 2.25)	1.27 (0.91, 1.77)^6^
Antenatal maternal alcohol use	1.30 (0.93, 1.82)	‐
Lower risk	REFERENCE	
Moderate risk	1.62 (1.02, 2.58)	‐
Higher risk	1.30 (0.64, 2.67)	‐
Family history asthma	1.28 (0.58, 2.83)	‐
Household income per month (South African Rand [ZAR])		
<R1000	REFERENCE	‐
R1000‐R5000	0.89 (0.69, 1.15)	‐
>R5000	0.92 (0.63, 1.33)	‐
Education		
Primary	REFERENCE	‐
Secondary	0.93 (0.59, 1.47)	‐
Completed secondary	0.76 (0.47, 1.22)	‐
Any tertiary	0.89 (0.47, 1.67)	‐
SES quartile		
Lowest	REFERENCE	‐
Moderately‐low	1.23 (0.88, 1.71)	‐
Moderately‐high	0.94 (0.67, 1.32)	‐
Highest	0.78 (0.56, 1.10)	‐
Sex—Female	0.83 (0.66, 1.05)	‐
Gestational age	0.98 (0.94, 1.02)	0.95 (0.91, 0.99)^6^
Preterm	1.19 (0.87, 1.63)	‐
Birth weight Z‐score	0.85 (0.76, 0.94)	0.84 (0.75, 0.95)^6^
Initiated breastfeeding	0.98 (0.66, 1.44)	‐
Duration of exclusive breastfeeding	0.94 (0.89, 1.00)	‐
DTaP‐IPV‐HIB 6‐10 weeks	1.23 (0.20, 7.39)	‐
PCV13 6‐10 weeks	0.62 (0.14, 2.78)	‐
BCG vaccine at birth	1.02 (0.23, 4.57)	‐
Site—TC Newman	1.63 (1.29, 2.07)	1.48 (1.09, 2.00)^6^
Child follow up time	1.08 (1.06, 1.10)	1.08 (1.06, 1.10)^6^

Multivariable models notation:

^1^ *N* = 983. Model 1: Antenatal maternal depression, maternal smoking, number of household smokers, gestational age, birthweight, site, follow‐up time.

^2^ *N* = 984. Model 2: Antenatal maternal psychological distress, maternal smoking, number of household smokers, gestational age, birthweight, site, follow‐up time.

^3^ *N* = 984. Model 3: Antenatal maternal IPV, maternal smoking, number of household smokers, gestational age, birthweight, site, follow‐up time.

^4^ *N* = 990. Model 4: Maternal childhood trauma, maternal smoking, number of household smokers, gestational age, birthweight, site, follow‐up time.

^5^ *N* = 982. Model 5: Antenatal maternal PTSD, maternal smoking, number of household smokers, gestational age, birthweight, site, follow‐up time.

^6^ *N* = 1122. Model 6: Maternal smoking, number of household smokers, gestational age, birthweight, site, follow‐up time.

However, in both the univariate and multivariable models, no psychosocial risk factors were associated with at least one episode of child wheeze.

#### 3.2.2 Antenatal psychosocial risk factors and recurrent child wheeze

When recurrent wheeze was considered as the outcome, antenatal psychological distress (OR = 1.58, 95%CI: 1.06–2.35) was found significant when considered independently, Table 4.

**Table 4:** Logistic regression: Antenatal psychosocial exposures and recurrent child wheeze

Predictors of recurrent child wheeze, with antenatal psychosocial risk factors as primary exposures		
Variable	Univariate OR (95%CI)	Multivariable OR (95%CI)
Antenatal maternal depression	1.35 (0.92, 1.97)	1.17 (0.77, 1.77)^7^
Antenatal maternal psychological distress	1.58 (1.06, 2.35)	1.42 (0.92, 2.19)^8^
Antenatal maternal IPV	1.35 (0.95, 1.91)	1.16 (0.79, 1.71)^9^
Antenatal maternal childhood trauma	1.34 (0.95, 1.90)	1.04 (0.71, 1.52)^10^
Antenatal maternal PTSD		
No PTSD exposure		‐
Suspect PTSD	1.26 (0.76, 2.08)	1.86 (1.08, 3.20)^11^
Trauma exposed	0.96 (0.57, 1.60)	1.09 (0.63, 1.90)^11^
Maternal age at delivery	0.99 (0.96, 1.02)	‐
Maternal HIV status	0.93 (0.62, 1.39)	‐
Maternal smoking	2.48 (1.77, 3.50)	1.78 (1.14, 2.78)^12^
Number of household smokers		
None	REFERENCE	‐
1 person	1.57 (1.04, 2.38)	1.06 (0.67, 1.70)^12^
2 or more people	1.88 (1.24, 2.84)	1.03 (0.63, 1.70)^12^
Antenatal maternal alcohol use		
Lower risk	REFERENCE	‐
Moderate risk	2.55 (1.49, 4.36)	1.94 (1.07, 3.51)^12^
Higher risk	1.32 (0.51, 3.44)	1.22 (0.44, 3.41)^12^
Family history asthma	1.37 (0.48, 3.89)	‐
Household income per month (South African Rand [ZAR])		
<R1000	REFERENCE	‐
R1000‐R5000	0.91 (0.64, 1.31)	‐
>R5000	1.16 (0.71, 1.90)	‐
Education		
Primary	REFERENCE	‐
Secondary	0.79 (0.44, 1.42)	‐
Completed secondary	0.65 (0.35, 1.20)	‐
Any tertiary	0.44 (0.17, 1.14)	‐
SES quartile		
Lowest	REFERENCE	‐
Moderately‐low	1.53 (0.97, 2.41)	‐
Moderately‐high	1.14 (0.72, 1.82)	‐
Highest	0.69 (0.42, 1.15)	‐
Sex—Female	0.53 (0.38, 0.74)	0.59 (0.41, 0.85)^12^
Gestational age	0.95 (0.90, 1.00)	0.91 (0.85, 0.98)^12^
Preterm	1.38 (0.91, 2.08)	‐
Birth weight Z‐score	0.86 (0.74, 0.99)	0.86 (0.72, 1.03)^12^
Initiated breastfeeding	1.37 (0.75, 2.49)	‐
Duration of exclusive breastfeeding	0.93 (0.85, 1.01)	‐
DTaP‐IPV‐HIB 6‐10 weeks	‐	‐
PCV13 6‐10 weeks	0.99 (0.10, 9.62)	‐
BCG vaccine at birth	0.59 (0.11, 3.27)	‐
Site—TC Newman	2.12 (1.53, 2.95)	1.60 (1.01, 2.53)^12^
Child follow up time	1.09 (1.06, 1.12)	1.08 (1.05, 1.12)^12^

Multivariable models notation:

^7^ *N* = 731. model 7: Antenatal maternal depression, maternal smoking, number of household smokers, antenatal maternal alcohol use, sex, gestational age, birthweight, site, follow‐up time.

^8^ *N* = 731. model 8: Antenatal maternal psychological distress, maternal smoking, number of household smokers, antenatal maternal alcohol use, sex, gestational age, birthweight, site, follow‐up time.

^9^ *N* = 731. model 9: Antenatal maternal IPV, maternal smoking, number of household smokers, antenatal maternal alcohol use, sex, gestational age, birthweight, site, follow‐up time.

^10^ *N* = 735. model 10: Maternal childhood trauma, maternal smoking, number of household smokers, antenatal maternal alcohol use, sex, gestational age, birthweight, site, follow‐up time.

^11^ *N* = 730. model 11: Antenatal maternal PTSD, maternal smoking, number of household smokers, antenatal maternal alcohol use, sex, gestational age, birthweight, site, follow‐up time.

^12^ *N* = 735. model 12: Maternal smoking, number of household smokers, antenatal maternal alcohol use, sex, gestational age, birthweight, site, follow‐up time.

However, in the multivariable analysis, none of the key antenatal psychosocial exposures, were associated with recurrent wheeze episodes. The multivariable models adjusted for antenatal maternal smoking, number of postnatal household smokers, antenatal alcohol use, gestation age, birth weight, sex, and clinic attended.

#### 3.2.3 Postnatal psychosocial risk factors and child wheeze

The 6‐month postnatal data was used to build the postnatal models, as outcomes at all the scheduled visits were highly correlated (Supplementary Table S4). In addition, a high proportion of wheezing episodes (49%) also took place within the first 6 months of life, Table 1.

The socio‐demographics of those who attended and did not attend the 6‐month psychosocial visit showed similar characteristics between the two groups, Supplementary Table S5. However, there was a higher number of smokers in those that attended the visit relative to those that did not attend. In addition, a higher proportion of those attending the visit were from TC Newman. Psychosocial risk factors measured antenatally and at the 12‐month postnatal visit were also compared between those attending and not attending the 6‐month psychosocial visit. There was no significant difference found in key exposures investigated (Supplementary Table S5) when comparing antenatal and postnatal psychosocial risk factors between those included and excluded from postnatal analyses.

#### 3.2.4 Postnatal psychosocial risk factors and presence of child wheeze

Maternal postnatal psychological distress and IPV were found to be significantly associated with child wheeze, when considered independently.

In the multivariable models, exposure to postnatal maternal psychological distress resulted in a twofold increased odds of developing at least one wheeze episode compared to those not exposed, while holding the other covariates constant (adjusted OR = 2.09, 95%CI, 1.16‐3.77). Postnatal maternal IPV exposure increased the odds of developing at least one episode of child wheeze by 63% (adjusted OR = 1.63, 95%CI, 1.13‐2.34) (Table 5).

**Table 5:** Logistic regression: postnatal psychosocial exposures and at least one episode of child wheeze

Predictors of at least one episode of child wheeze, with postnatal psychosocial risk factors as primary exposures		
Variable	Univariate OR (95%CI)	Multivariable OR (95%CI)
Postnatal maternal depression	1.51 (0.98,2.33)	1.38 (0.88, 2.17)^13^
Postnatal maternal psychological distress	2.52 (1.44, 4.41)	2.09 (1.16, 3.77)^14^
Postnatal maternal IPV	1.82 (1.29, 2.58)	1.63 (1.13, 2.34)^15^
Postnatal maternal PTSD		
No PTSD exposure	REFERENCE	‐
Suspect PTSD	1.13 (0.48, 2.67)	1.35 (0.54, 3.37)^16^
Trauma exposed	2.05 (0.72, 5.84)	2.23 (0.75, 6.58)^16^
Maternal age at delivery	0.99 (0.97, 1.01)	‐
Maternal HIV status	1.02 (0.76, 1.35)	‐
Maternal smoking	1.68 (1.29, 2.18)	1.26 (0.91, 1.74)^17^
Number of household smokers		
None	REFERENCE	‐
1 person	1.47 (1.10, 1.96)	1.27 (0.94, 1.73)^17^
2 or more people	1.69 (1.26, 2.27)	1.27 (0.91, 1.77)^17^
Antenatal maternal alcohol use	1.30 (0.93, 1.82)	‐
Lower risk	REFERENCE	
Moderate risk	1.62 (1.02, 2.58)	‐
Higher risk	1.30 (0.64, 2.67)	‐
Family history asthma	1.28 (0.58, 2.83)	‐
Household income per month (South African Rand [ZAR])		
<R1000	REFERENCE	‐
R1000‐R5000	0.89 (0.69, 1.15)	‐
>R5000	0.92 (0.63, 1.33)	‐
Education		
Primary	REFERENCE	‐
Secondary	0.93 (0.59, 1.47)	‐
Completed secondary	0.76 (0.47, 1.22)	‐
Any tertiary	0.89 (0.47, 1.67)	‐
SES quartile		
Lowest	REFERENCE	‐
Moderately‐low	1.23 (0.88, 1.71)	‐
Moderately‐high	0.94 (0.67, 1.32)	‐
Highest	0.78 (0.56, 1.10)	‐
Sex—Female	0.83 (0.66, 1.05)	‐
Gestational age	0.98 (0.94, 1.02)	0.95 (0.91, 0.99)^17^
Preterm	1.19 (0.87, 1.63)	‐
Birth weight Z‐score	0.85 (0.76, 0.94)	0.84 (0.75, 0.95)^17^
Initiated breastfeeding	0.98 (0.66, 1.44)	‐
Duration of exclusive breastfeeding	0.94 (0.89, 1.00)	‐
DTap‐IPV‐HIB 6‐10 weeks	1.23 (0.20, 7.39)	‐
PCV13 6‐10 weeks	0.62 (0.14, 2.78)	‐
BCG vaccine at birth	1.02 (0.23, 4.57)	‐
Site—TC Newman	1.63 (1.29, 2.07)	1.48 (1.09, 2.00)^17^
Child follow up time	1.08 (1.06, 1.10)	1.08 (1.06, 1.10)^17^

Multivariable models notation:

^13^ *N* = 641. Model 13: Postnatal maternal depression, maternal smoking, number of household smokers, gestational age, birthweight, site, follow‐up time.

^14^ *N* = 639. Model 14: Postnatal maternal psychological distress, maternal smoking, number of household smokers, gestational age, birthweight, site, follow‐up time.

^15^ *N* = 639. Model 15: Postnatal maternal IPV, maternal smoking, number of household smokers, gestational age, birthweight, site, follow‐up time.

^16^ *N* = 251. Model 16: Postnatal maternal PTSD, maternal smoking, number of household smokers, gestational age, birthweight, site, follow‐up time.

^17^ *N* = 1122. Model 17: Maternal smoking, number of household smokers, gestational age, birthweight, site, follow‐up time.

#### 3.2.5 Postnatal psychosocial risk factors and recurrent child wheeze

From Table 6, the odds of experiencing recurrent wheezing episodes increased by 96% in those whose mothers displayed postnatal depressive symptoms, compared to those whose mothers did not, when considered independently. However, stronger associations were observed when psychological distress (OR = 3.12, 95%CI, 1.61‐6.02) and IPV (OR = 2.68, 95%CI, 1.73‐4.15) were considered.

**Table 6:** Logistic regression: postnatal psychosocial exposures and recurrent child wheeze

Predictors of recurrent child wheeze, with postnatal psychosocial risk factors as primary exposures		
Variable	Univariate OR (95%CI)	Multivariable OR (95%CI)
Postnatal maternal depression	1.96 (1.15, 3.33)	1.48 (0.82, 2.66)^18^
Postnatal maternal psychological distress	3.12 (1.61, 6.02)	2.26 (1.06, 4.81)^19^
Postnatal maternal IPV	2.68 (1.73, 4.15)	2.20 (1.35, 3.61)^20^
Postnatal maternal PTSD		
No PTSD exposure	REFERENCE	‐
Suspect PTSD	1.33 (0.44, 4.02)	1.61 (0.40, 6.46)^21^
Trauma exposed	1.59 (0.38, 6.69)	1.01 (0.15, 6.94)^21^
Maternal age at delivery	0.99 (0.96, 1.02)	‐
Maternal HIV status	0.93 (0.62, 1.39)	‐
Maternal smoking	2.48 (1.77, 3.50)	1.78 (1.14, 2.78)^22^
Number of household smokers		
None	REFERENCE	‐
1 person	1.57 (1.04, 2.38)	1.06 (0.67, 1.70)^22^
2 or more people	1.88 (1.24, 2.84)	1.03 (0.63, 1.70)^22^
Antenatal maternal alcohol use		
Lower risk	REFERENCE	‐
Moderate risk	2.55 (1.49, 4.36)	1.94 (1.07, 3.51)^22^
Higher risk	1.32 (0.51, 3.44)	1.22 (0.44, 3.41)^22^
Family history asthma	1.37 (0.48, 3.89)	‐
Household income per month (South African Rand [ZAR])		
<R1000	REFERENCE	‐
R1000‐R5000	0.91 (0.64, 1.31)	‐
>R5000	1.16 (0.71, 1.90)	‐
Education		
Primary	REFERENCE	‐
Secondary	0.79 (0.44, 1.42)	‐
Completed secondary	0.65 (0.35, 1.20)	‐
Any tertiary	0.44 (0.17, 1.14)	‐
SES quartile		
Lowest	REFERENCE	‐
Moderately‐low	1.53 (0.97, 2.41)	‐
Moderately‐high	1.14 (0.72, 1.82)	‐
Highest	0.69 (0.42, 1.15)	‐
Sex—Female	0.53 (0.38, 0.74)	0.59 (0.41, 0.85)^22^
Gestational age	0.95 (0.90, 1.00)	0.91 (0.85, 0.98)^22^
Preterm	1.38 (0.91, 2.08)	‐
Birth weight Z‐score	0.86 (0.74, 0.99)	0.86 (0.72, 1.03)^22^
Initiated breastfeeding	1.37 (0.75, 2.49)	‐
Duration of exclusive breastfeeding	0.92 (0.84, 1.02)	‐
DTap‐IPV‐HIB 6‐10 weeks	‐	‐
PCV13 6‐10 weeks	0.99 (0.10, 9.62)	‐
BCG vaccine at birth	0.59 (0.11, 3.27)	‐
Site—TC Newman	2.12 (1.53, 2.95)	1.60 (1.01, 2.53)^22^
Child follow up time	1.09 (1.06, 1.12)	1.08 (1.05, 1.12)^22^

Multivariable models notation:

^18^ *N* = 421. Model 18: Postnatal maternal depression, maternal smoking, number of household smokers, antenatal maternal alcohol use, sex, gestational age, birthweight, site, follow‐up time.

^19^ *N* = 421. Model 19: Postnatal maternal psychological distress, maternal smoking, number of household smokers, antenatal maternal alcohol use, sex, gestational age, birthweight, site, follow‐up time.

^20^ *N* = 420. Model 20: Postnatal maternal IPV, maternal smoking, number of household smokers, antenatal maternal alcohol use, sex, gestational age, birthweight, site, follow‐up time.

^21^ *N* = 156. Model 21: Postnatal maternal PTSD, maternal smoking, number of household smokers, antenatal maternal alcohol use, sex, gestational age, birthweight, site, follow‐up time.

^22^ *N* = 735. Model 22: Maternal smoking, number of household smokers, antenatal maternal alcohol use, sex, gestational age, birthweight, site, follow‐up time.

In the multivariable models, psychological distress and IPV were significantly associated with recurrent wheeze. Children whose mothers suffered from postnatal psychological distress had 2.3‐fold increased odds in experiencing recurrent wheezing episodes, compared to those not exposed (adjusted OR = 2.26, 95%CI: 1.06‐4.81). In addition, postnatal IPV exposure also had a substantial influence on recurrent wheeze (adjusted OR = 2.20, 95%CI, 1.35‐3.61).

## 4 DISCUSSION

In this peri‐urban, low‐income area of South Africa, postnatal maternal psychological distress and IPV were strongly associated with early childhood wheezing, after adjusting for key clinical and socio‐demographic exposures. Surprisingly, none of the antenatal psychosocial risk factors were associated with child wheezing. These findings suggest that postnatal maternal psychosocial risk factors increase the risk of early childhood wheeze or recurrent wheeze in a LMIC context.

Several known exposures were also identified as being associated with early wheezing including ETS exposure, low birth weight and prematurity, all of which are well‐known risk factors for child wheeze; thus, these associations extend into LMIC settings. The effects of passive smoke, as well as exposure to ETS are widely known,^31^ and this was confirmed, as maternal smoking resulted in a twofold increased odds of recurrent child wheeze. Additional household smokers also placed a child at increased risk for wheezing.

However, antenatal maternal depression and IPV were not associated with either one episode of wheezing or recurrent child wheeze. This is inconsistent with previous literature, which found that antenatal psychosocial risk factor exposure predicted child wheeze and asthma diagnosis. Reyes et al^4^ found that antenatal psychological distress predicted recurrent wheeze in early stages of life, in both independent and adjusted models. In addition, Ramratnam et al.,^5^ and Mathilda Chiu et al.,^6^ found that antenatal depression was significantly associated with recurrence of child wheeze. As these studies were conducted in HIC, urban, low‐income, and genetically predisposed children, they attributed the associations to biological mechanisms, such as the release of stress hormones, which affect foetal development in utero.

Previous studies have found that antenatal maternal psychosocial exposure impacts birth weight as well as lung development, which may lead to airway obstruction.^13^, ^32^ As this is the case, there could be an indirect link between antenatal maternal psychosocial health and child wheeze. In this study, no direct link was found between antenatal depression or IPV and child wheeze; however, birth weight, and gestational age were found to be confounding variables in the relationship between maternal psychosocial exposure and the onset of child wheeze. Thus, these antenatal psychosocial exposures may be impacting on child wheeze through these biological mechanisms.

When postnatal exposures were considered, maternal psychological distress, as well IPV exposure, were strongly associated with the child experiencing at least one episode of wheezing during the 24‐month period, as well as recurrent wheeze episodes. These results strengthen the findings of several studies reporting on the relationship between maternal psychosocial risk factors and childhood wheeze and asthma,^4^, ^8^, ^9^, ^10^, ^11^, ^12^, ^13^, ^33^ and extend the association into a LMIC context.

Potential mechanisms suggested for the association with postnatal maternal psychological distress or IPV exposure and child wheeze have included impaired maternal‐child relationship and a mother's inability to provide care for her child.^34^ A mother that is either exposed to IPV or suffering from mental illness is also more likely to engage in harmful behaviors such as drinking or smoking. This was found to be true in the context of this study, as maternal smoking and drinking habits were found to be associated with postnatal psychosocial risk factors.

As antenatal and postnatal maternal psychosocial risk factors were closely correlated in this study, the effect of postnatal exposure may represent cumulative exposure beginning antenatally and continuing through the postnatal period. An example of this could be biological mechanisms, such as higher cortisol levels in the children due to increased stress hormones being passed from mother to child in‐utero.^35^ At risk children may be less able to respond to stressors, and thus more prone to wheezing or asthma diagnosis later in life.^36^ This may occur, for example, if the mother‐child interaction is disturbed through maternal psychosocial risk factor exposures; such alterations have been observed in children whose mothers are exposed to postnatal IPV.^36^ Due to this study's findings, biological mechanisms should be investigated in these children to better understand the relationship between maternal psychosocial risk factor exposure and child wheezing in a LMIC context.

The main limitation was the use of one time‐point, the 6‐month scheduled visit, to represent the postnatal exposure of psychosocial risk factors. However, as the measurable outcomes of the psychosocial risk factors were correlated over time, it was deemed appropriate to use one time‐point. Further research into the subject should consider a longitudinal analysis to investigate the impact of changing postnatal psychosocial risk factors on child wheeze.

An additional limitation is that a higher number of smokers were present in those attending the scheduled 6‐month psychosocial visits compared to those not attending the visit. This may overestimate the strength of association between postnatal psychosocial risk factors and child wheeze.

In previous studies, maternal or caregiver self‐report of wheeze was used to identify episodes, which may have resulted in an under‐ or over‐reporting of wheeze episodes. In the DCHS, respiratory illness symptoms including wheeze were prospectively and actively surveyed by members of the study team, which may improve accuracy of wheeze episode prevalence. The prospective nature of the study provides another strength, especially in terms of antenatal psychosocial measures in relation to child wheeze outcome, as this allows temporality to be considered.

In conclusion, postnatal psychological distress and IPV predicted the development and recurrence of child wheezing in the first 2 years of life. With increasing wheeze prevalence and severity in LMIC settings as well as resource‐limited mental health services, it is important to understand the psychosocial risk factors for child wheeze. Understanding how maternal mental health may influence children's respiratory health is an important step in developing effective interventions that lessen the burden of wheeze and asthma in LMIC, such as South Africa.

## CONFLICTS OF INTEREST

None.

## Supporting information

Additional Supporting Information may be found online in the supporting information tab for this article.

**Table S1**. Chi‐squared test of Independence (correlation) between antenatal and postnatal EPDS threshold
**Table S2**. Chi‐squared test of independence (correlation) between antenatal and postnatal SRQ20 threshold.
**Table S3**.Chi‐squared test of independence (correlation) between antenatal and postnatal IPV threshold.
**Table S4**. Chi‐squared test of independence (correlation) among psychosocial measures.
**Table S5**. Socio‐demographic comparison between those attending and not attending 6‐month psychosocial visit.

**Fig. S1**. Consort diagram of enrolment, lost to follow up and visit attended.
