# Supplementary material for: Associations between maternal mental health and early child wheezing in a South African birth cohort
Source: Pediatr Pulmonol. 2018 Apr 10;53(6):741–54. doi: 10.1002/ppul.24008 (PMC6001799; doi:10.1002/ppul.24008)
Supplement: Supplementary file 1 — Table S1. Chi‐squared test of Independence (correlation) between antenatal and postnatal EPDS threshold Table S2. Chi‐squared test of independence (correlation) between antenatal and postnatal SRQ20 threshold. Table S3.Chi‐squared test of independence (correlation) between antenatal and postnatal IPV threshold. Table S4. Chi‐squared test of independence (correlation) among psychosocial measures. Table S5. Socio‐demographic comparison between those attending and not attending 6‐month psychosocial visit. [file PPUL-53-741-s001.docx]

E-Table 1 Chi-squared test of Independence (correlation) between antenatal and postnatal EPDS threshold

| EPDS* Threshold^**^ | Odds ratios (P-value) | | | |
| --- | --- | --- | --- | --- |
|  | Antenatal | 6-10 weeks | 6-month | 12-month |
| 6-10 weeks | 4.66  (<0.0001) | - | - | - |
| 6-month | 4.75  (<0.0001) | 8.52  (<0.0001) | - | - |
| 12-month | 2.67  (<0.0001) | 4.86  (<0.0001) | 6.20  (<0.0001) | - |
| 18-month | 5.19  (<0.0001) | 3.90  (<0.0001) | 6.24  (<0.0001) | 8.49  (<0.0001) |

^* EPDS = Edinburgh Postnatal Depression Scale; used to determine presence of depression^

^**EPDS collected through to 18 months post-delivery^

E-Table 2 Chi-squared test of independence (correlation) between antenatal and postnatal SRQ20 threshold

| SRQ20 Threshold^*^ | Odds ratios (P-value) | | | | |
| --- | --- | --- | --- | --- | --- |
|  | Antenatal | 6-10 weeks | 6-month | 12-month | 18-month |
| 6-10 weeks | 5.81  (<0.0001) | - | - | - | - |
| 6-month | 8.16 (<0.0001) | 27.61 (<0.0001) | - | - | - |
| 12-month | 6.42 (<0.0001) | 18.66 (<0.0001) | 27.08 (<0.0001) | - | - |
| 18-month | 7.43 (<0.0001) | 20.93 (<0.0001) | 16.06 (<0.0001) | 30.21 (<0.0001) | - |
| 24-month | 7.23 (<0.0001) | 14.45 (<0.0001) | 25.07 (<0.0001) | 27.84 (<0.0001) | 43.03 (<0.0001) |

^*SRQ20 = Self-reporting Questionnaire 20-items; used to assess presence of psychological distress^

E-Table 3 Chi-squared test of independence (correlation) between antenatal and postnatal IPV threshold

| IPV Threshold^*^ | Odds ratios (P-value) | | | | |
| --- | --- | --- | --- | --- | --- |
|  | Antenatal | 6-10 weeks | 6-month | 12-month | 18-month |
| 6-10 weeks | 5.76 (<0.0001) | - | - | - | - |
| 6-month | 5.89 (<0.0001) | 10.22 (<0.0001) | - | - | - |
| 12-month | 5.06 (<0.0001) | 4.77 (<0.0001) | 11.07 (<0.0001) | - | - |
| 18-month | 4.01 (<0.0001) | 4.31 (<0.0001) | 6.30 (<0.0001) | 6.04 (<0.0001) | - |
| 24-month | 3.29 (<0.0001) | 4.50 (<0.0001) | 5.87 (<0.0001) | 5.50 (<0.0001) | 6.34 (<0.0001) |

^*IPV = Intimate Partner Violence^

E-Table 4 Chi-squared test of independence (correlation) among psychosocial measures

| Psychosocial measure | Odds ratios (P-value) | | | | |
| --- | --- | --- | --- | --- | --- |
|  | EPDS antenatal | EPDS postnatal | SRQ20 antenatal | SRQ20 postnatal | IPV antenatal |
| EPDS postnatal | 4.75 (<0.0001) | - | - | - | - |
| SRQ20 antenatal | 7.33 (<0.0001) | 2.97 (<0.0001) | - | - | - |
| SRQ20 postnatal | 5.81 (<0.0001) | 23.31 (<0.0001) | 8.16 (<0.0001) | - | - |
| IPV antenatal | 2.69 (<0.0001) | 2.83 (<0.0001) | 2.73 (<0.0001) | 4.62 (<0.0001) | - |
| IPV antenatal | 1.99 (<0.0001) | 2.55 (<0.0001) | 2.51 (<0.0001) | 5.86 (<0.0001) | 5.89 (<0.0001) |

^EPDS antenatal = maternal antenatal depression measurement; EPDS postnatal = maternal postnatal depression measurement^

^SRQ20 antenatal = maternal antenatal psychological distress measurement; SRQ20 postnatal = maternal postnatal psychological distress measurement^

^IPV antenatal = maternal antenatal intimate partner violence measurement; IPV postnatal = maternal postnatal intimate partner violence measurement.^

Contingency table were constructed to investigate whether scheduled psychosocial measures were correlated over time. E-Table 1 – E-Table 4, considered the maternal depression, psychological distress and IPV measures independently. The contingency table below display odds ratios (OR) and p-value, based on Chi-squared Test of independent tests. With the large odds ratios observed across E-Table 1 – E-Table 4, there is sufficient evidence to suggest that the psychosocial risk factor measures are highly associated with other over time. This is further evident from the p-values (<0.05), that suggest that there are significant relationships that exist between the psychosocial risk factor measures over time.

E-Table 5, considered whether correlation was present among the psychosocial risk factors. Based on the high odds ratios displayed, there is evidence to suggest that maternal depression, psychological distress and IPV exposure are correlated with one another.

In order to justify the use of 6-month psychosocial data to represent postnatal exposure, demographic information, as well as the psychosocial risk factor exposures antentally and 12 months postpartum, were compared between those who attended and those who did not attend the 6-month psychosocial visit.

Based on E-Table 5, there were similar household income and education levels between the two groups. In addition, alcohol consumption was similar between those attending and not attending the 6-month psychosocial visit. The birth characteristics were also similar, as the median birthweight and gestation age was 3.09kg and 39 weeks respectively. However, maternal smoking did differ between the two groups, as 33% of those who attended the 6-month psychosocial visit smoked, while only 19% of those that did not attend smoked.

When considering the antenatal psychosocial risk factors, maternal depression and psychological distress were similar between the two groups. However, antenatal IPV was statistically different between the two groups. Although, all the psychosocial risk factors, measured at the 12-month visit, were similar between the two groups.

As the majority of characteristics were similar between the two groups, utilising the 6-month psychosocial data as a proxy for postnatal exposure was appropriate.

E-Table 5: Socio-demographic comparison between those attending and not attending 6-month psychosocial visit

| Variable | Attended 6-month psychosocial visit (N=646) | Did not attend 6-month psychosocial visit (N=497) | P-value |
| --- | --- | --- | --- |
| Site - TC Newman | 319 (49.8%) | 190 (38.2%) | <0.0001 |
| Female | 313 (48.5%) | 241 (48.5%) | 0.990 |
| Household income per month (South African Rand [ZAR]) | | | |
| < R1000 | 255 (39.5%) | 166 (33.7%) | 0.103 |
| R1000 – R5000 | 311 (48.1%) | 253 (51.3%) | - |
| > R5000 | 80 (12.4%) | 74 (15.0%) | - |
| Education | | | |
| Primary | 46 (7.1%) | 40 (8.0%) | 0.101 |
| Secondary | 365 (56.5%) | 244 (49.1%) | - |
| Completed secondary | 197 (30.5%) | 178 (35.8%) | - |
| Any tertiary | 38 (5.9%) | 35 (7.0%) | - |
| Socio-economic status quartile | | | |
| Lowest quartile | 157 (24.3%) | 117 (23.4%) | 0.030 |
| Low - moderate | 175 (27.1%) | 121 (24.3%) | - |
| High - moderate | 175 (27.1%) | 115 (23.1%) | - |
| Highest quartile | 139 (21.5%) | 144 (29.0%) | - |
| Maternal or household characteristics | | | |
| Maternal age at delivery | 26.35 (22.21 – 31.01) | 25.92 (22.32 – 31.12) | 0.489 |
| Maternal smoking | 211 (32.7%) | 95 (19.1%) | <0.0001 |
| Other household smokers | | | |
| One smoker | 242 (37.5%) | 154 (31.0%) | <0.0001 |
| Two or more smokers | 243 (37.6%) | 126 (25.4%) | - |
| Antenatal maternal alcohol use |  |  |  |
| Lower risk | 513 (89.1%) | 373 (89.0%) | 0.712 |
| Moderate risk | 47 (8.2%) | 31 (7.4%) | - |
| Higher risk | 16 (2.8%) | 15 (3.6%) | - |
| Maternal HIV | 138 (21.4%) | 110 (22.1%) | 0.714 |
| Family history of asthma | 13 (2.4%) | 12 (1.9%) | 0.645 |
| Birth characteristics | | | |
| Gestation (weeks) | 39 (37.5 – 40) | 39 (38 – 40) | 0.836 |
| Prematurity (< 37 weeks) | 99 (15.3%) | 95 (19.1%) | 0.091 |
| Birthweight (kg) | 3.09 (2.70 – 3.42) | 3.09 (2.73 – 3.41) | 0.917 |
| Feeding choice | | | |
| Initiated breastfeeding | 603 (93.3%) | 444 (89.3%) | 0.015 |
| Exclusive breastfeeding months | 1.2 (0.46 – 3) | 0.92 (0.46 – 2.76) | 0.116 |
| Psychosocial measures at antenatal and 12-month visit | | | |
| Antenatal maternal depression | 147/580 (25.3%) | 90/414 (21.6%) | 0.188 |
| Postnatal maternal depression (12-month visit) | 79/483 (16.4%) | 37/234 (15.8%) | 0.853 |
| Antenatal maternal psychological distress | 120/580 (20.7%) | 81/411 (19.6%) | 0.705 |
| Postnatal maternal psychological distress (12-month visit) | 43/496 (8.7%) | 27/258 (10.5%) | 0.420 |
| Antenatal maternal IPV* | 217/579 (37.5.8%) | 115/411 (28.0%) | 0.002 |
| Postnatal maternal IPV (12-month visit) | 130/488 (26.6%) | 69/254 (27.2%) | 0.878 |

^*IPV = Intimate Partner Violence^
