# Supplementary figures and images for: Associations between maternal mental health and early child wheezing in a South African birth cohort
Source: Pediatr Pulmonol. 2018 Apr 10;53(6):741–54. doi: 10.1002/ppul.24008 (PMC6001799; doi:10.1002/ppul.24008)

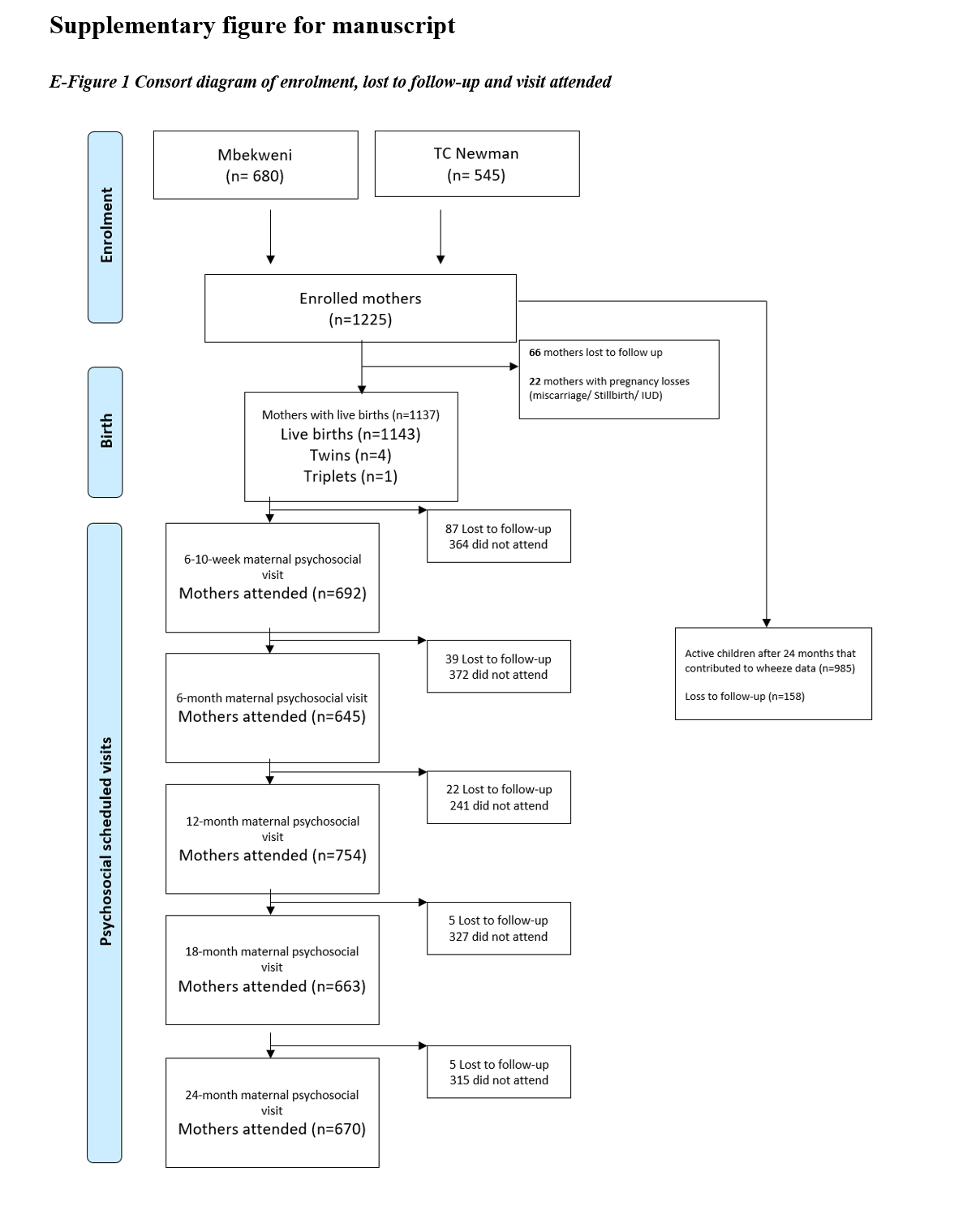

Supplement: Supplementary file 2 — Fig. S1. Consort diagram of enrolment, lost to follow up and visit attended. [file PPUL-53-741-s002.TIF]
